# SoilGrids1km — Global Soil Information Based on Automated Mapping

**DOI:** 10.1371/journal.pone.0105992

**Published:** 2014-08-29

**Authors:** Tomislav Hengl, Jorge Mendes de Jesus, Robert A. MacMillan, Niels H. Batjes, Gerard B. M. Heuvelink, Eloi Ribeiro, Alessandro Samuel-Rosa, Bas Kempen, Johan G. B. Leenaars, Markus G. Walsh, Maria Ruiperez Gonzalez

**Affiliations:** 1 ISRIC — World Soil Information, Wageningen, the Netherlands; 2 LandMapper Environmental Solutions Inc., Edmonton, Canada; 3 Wageningen University, Wageningen, the Netherlands; 4 Federal Rural University of Rio de Janeiro, Rio de Janeiro, Brazil; 5 The Earth Institute, Columbia University, New York, New York, United States of America, and Selian Agricultural Research Inst., Arusha, Tanzania; DOE Pacific Northwest National Laboratory, United States of America

## Abstract

**Background:**

Soils are widely recognized as a non-renewable natural resource and as biophysical carbon sinks. As such, there is a growing requirement for global soil information. Although several global soil information systems already exist, these tend to suffer from inconsistencies and limited spatial detail.

**Methodology/Principal Findings:**

We present SoilGrids1km — a global 3D soil information system at 1 km resolution — containing spatial predictions for a selection of soil properties (at six standard depths): soil organic carbon (g kg−1), soil pH, sand, silt and clay fractions (%), bulk density (kg m−3), cation-exchange capacity (cmol+/kg), coarse fragments (%), soil organic carbon stock (t ha−1), depth to bedrock (cm), World Reference Base soil groups, and USDA Soil Taxonomy suborders. Our predictions are based on global spatial prediction models which we fitted, per soil variable, using a compilation of major international soil profile databases (ca. 110,000 soil profiles), and a selection of ca. 75 global environmental covariates representing soil forming factors. Results of regression modeling indicate that the most useful covariates for modeling soils at the global scale are climatic and biomass indices (based on MODIS images), lithology, and taxonomic mapping units derived from conventional soil survey (Harmonized World Soil Database). Prediction accuracies assessed using 5–fold cross-validation were between 23–51%.

**Conclusions/Significance:**

SoilGrids1km provide an initial set of examples of soil spatial data for input into global models at a resolution and consistency not previously available. Some of the main limitations of the current version of SoilGrids1km are: (1) weak relationships between soil properties/classes and explanatory variables due to scale mismatches, (2) difficulty to obtain covariates that capture soil forming factors, (3) low sampling density and spatial clustering of soil profile locations. However, as the SoilGrids system is highly automated and flexible, increasingly accurate predictions can be generated as new input data become available. SoilGrids1km are available for download via http://soilgrids.org under a Creative Commons Non Commercial license.

## Introduction

There is increasing recognition of the urgent need to improve the quality, quantity and spatial detail of information about soils to respond to challenges presented by growing pressures on soils to support a large variety of critical functions [1]–[4]. Arrouays et al. [3] argue that existing soils information is not well suited to addressing vital questions related to mapping, monitoring or modelling soil processes that are driven by fluxes or changes in soils of water, nutrients, carbon, solutes or energy. Conventional models of soil variation describe variation in the horizontal dimension using polygons comprising classes of named soils [5]. In the vertical dimension, variation is described in terms of classes of horizons or layers that vary in their properties, thickness and depth. These conceptual models of discrete variation of classes of soil in horizontal and vertical directions are not well suited for use in many of the (global) simulation models and decision making systems currently used to describe and interpret soil functions and processes, such as supporting crop growth modelling, modelling hydrological and climatological processes, soil carbon dynamics or erosion [2], [5]. Most modern spatial models that require information about soils as an input need accurate numerical information about continuous variation in soil properties. Models also require input data layers that are complete, consistent and as correct and current as possible. These requirements are not well met by current sources of soils information, especially sources of global extent.

Soil is probably one of the least well described thematic layers at the global scale, and existing global soil maps are often of undocumented or unknown accuracy [5]. At the moment, only coarse scale soil maps of the world are available at an effective resolution of about ∼20 km [10]. The most commonly used global soil maps include [2], [5]: Harmonized World Soil Database (HWSD) [11], USGS-produced soil property maps (http://soils.usda.gov/use/worldsoils/mapindex/) and ISRIC-WISE based soil property maps [12].

**Figure 1:**
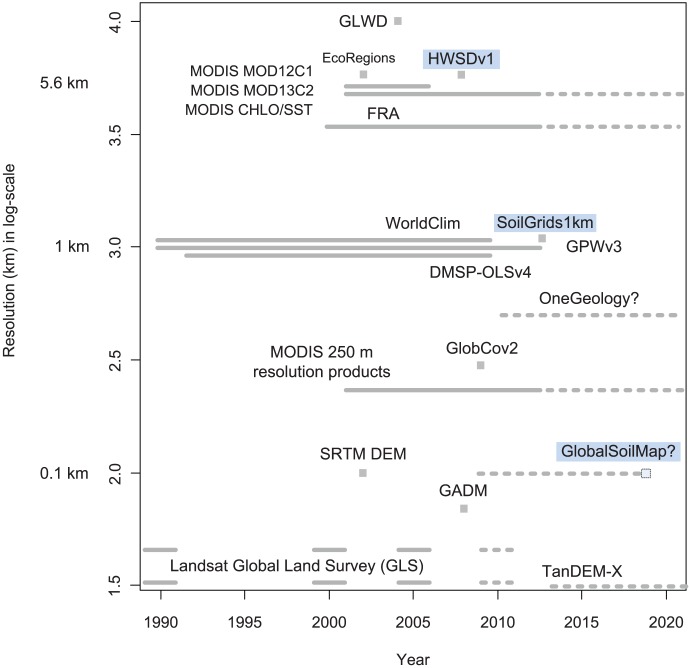
Spatial resolution and temporal coverage/publication time of some widely used global environmental data layers (global soil layers have been highlighted): GLWD — Global Lakes and Wetlands Database, HWSD — Harmonized World Soil Database, MOD12C1 — MODIS Land Cover Type Yearly L3, MOD13C2 — Vegetation Indices Monthly L3, CHLO/SST — MODIS Aqua Level-3 annual Chlorophyll/mid-IR Sea Surface Temperature, FRA — Forest Resources Assessment, GPW — Gridded Population of the World, DMSP-OLS — Nighttime Lights Time Series, GlobCov — Land Cover classes based on the MERIS FR images, GADM — Global Administrative Areas, TanDEM-X — Germany's topographic radar mission. Key agenda setters in the terms of production and dissemination of remote sensing and thematic environmental layers at the beginning of the 21st century include: NASA's MODIS (Moderate-resolution Imaging Spectroradiometer) and Landsat products — in terms of thematic content and usability [6]–[8], and Germany's TanDEM-X new global 12 m resolution DEM with ±2 m vertical accuracy [9]. Based on information retrieved on February 15th 2014. was produced using the Global Soil Information Facilities (GSIF), which was recently developed at ISRIC as a framework and platform to support widespread, open collaboration in the assembly, collation and production of global soil information.

While widely used and cited, these various coarse resolution soil maps tend to suffer from artefacts due to use of different soil mapping concepts between countries and regions, from variation in the underlying soil mapping scale (usually between 1∶0.5 M to 1∶5 M) and from differences in reliability of source data within and between continents [2], [5]. They can also not easily be updated with new information and often lack any measure of uncertainty, which is assumed to be significant. In summary, currently available global soil maps are not comparable in level of detail, spatial accuracy and usability with other global environmental layers such as global land cover and climatic products (Figure 1).

In this paper, we present and describe SoilGrids1km — a global 3D soil information system at 1 resolution — as a first response to the need for a new, consistent and coherent, global soil information. SoilGrids1km was produced using the Global Soil Information Facilities (GSIF), which was recently developed at ISRIC as a framework and platform to support widespread, open collaboration in the assembly, collation and production of global soil information.

## Materials and Methods

### Global Soil Information Facilities

ISRIC — World Soil Information has a mandate to serve the international community with information about the world's soil resources to help addressing major global issues. Over the last four years, in collaboration with a growing number of international partners and with a direct support from the Bill and Melinda Gates Foundation (AfSIS project; http://africasoils.net), ISRIC has been developing a cyberinfrastructure called Global Soil Information Facilities (GSIF).

GSIF has a particular emphasis on supporting the assembly and collation of geo-registered soil profile descriptions with associated analytical data, and on supporting the production of new maps of 3D continuous soil properties and soil classes at global to regional scales. GSIF consists of several components: data portals for assembling and hosting soil profile data and covariate data, software for global soil data analysis and mapping, and facilities for documenting data and methods and for automating workflows.

One of these components is “SoilGrids” — an automated system for global soil mapping. SoilGrids is an implementation of model-based geostatistics [13], [14] for the purpose of predicting soil properties (in 2D or 3D) and soil classes for a global soil mask (see further Figure 3c) using automated mapping. Automated mapping is the computer- aided generation of maps from point observations and covariate layers, with minimal human intervention, so that map updating is easy. In the context of geostatistical mapping, automated mapping implies that model fitting, prediction and visualization are run using fully automated and reproducible workflows [14], [15]. The current implementation of SoilGrids focuses on producing predictions at 1 km spatial resolution and for a selection of soil properties and classes of interest to modelers and to international organizations such as FAO, Intergovernmental Panel on Climate Change (IPCC), the Consultative Group on International Agricultural Research (CGIAR) and similar.

We have imagined GSIF as a crowd-sourcing system, largely inspired by systems such as OpenStreetMap, Geo-wiki [16] and the R Open Source environment for statistical computing [17]. In this context, GSIF follows the “Agile” approach to software/IT development [18] meaning that we support rapid development, integration of soil field data, output validation, and rapid publishing of results. A new development cycle with new outputs (in principle of improved accuracy) is implemented in succession within an automated processing framework until the desired target specifications have been reached.

### Input data for SoilGrids1km

The main input data sources for SoilGrids1km are global compilations of publicly available (shared) soil profile data and environmental layers at 1 km resolution; both are freely accessible via portals (http://worldsoilprofiles and http://www.worldgrids.org). The main sources of soil profile data used to produce the first version of SoilGrids1km are: the USA National Cooperative Soil Survey Soil Characterization database (http://ncsslabdatamart.sc.egov.usda.gov/) and profiles from the USA National Soil Information System (http://soils.usda.gov/technical/nasis/), LUCAS Topsoil Survey database [19], Africa Soil Profiles database [20], Mexican National soil profile database [21], Brazilian national soil profile database [22], Chinese soil profile database [23], and the soil profile archive from the Canadian Soil Information System [24]. Other significant sources of profile data used are: ISRIC-WISE [25], SOTER [26], SPADE [27], and Russian soil reference profiles [28].

**Figure 2:**
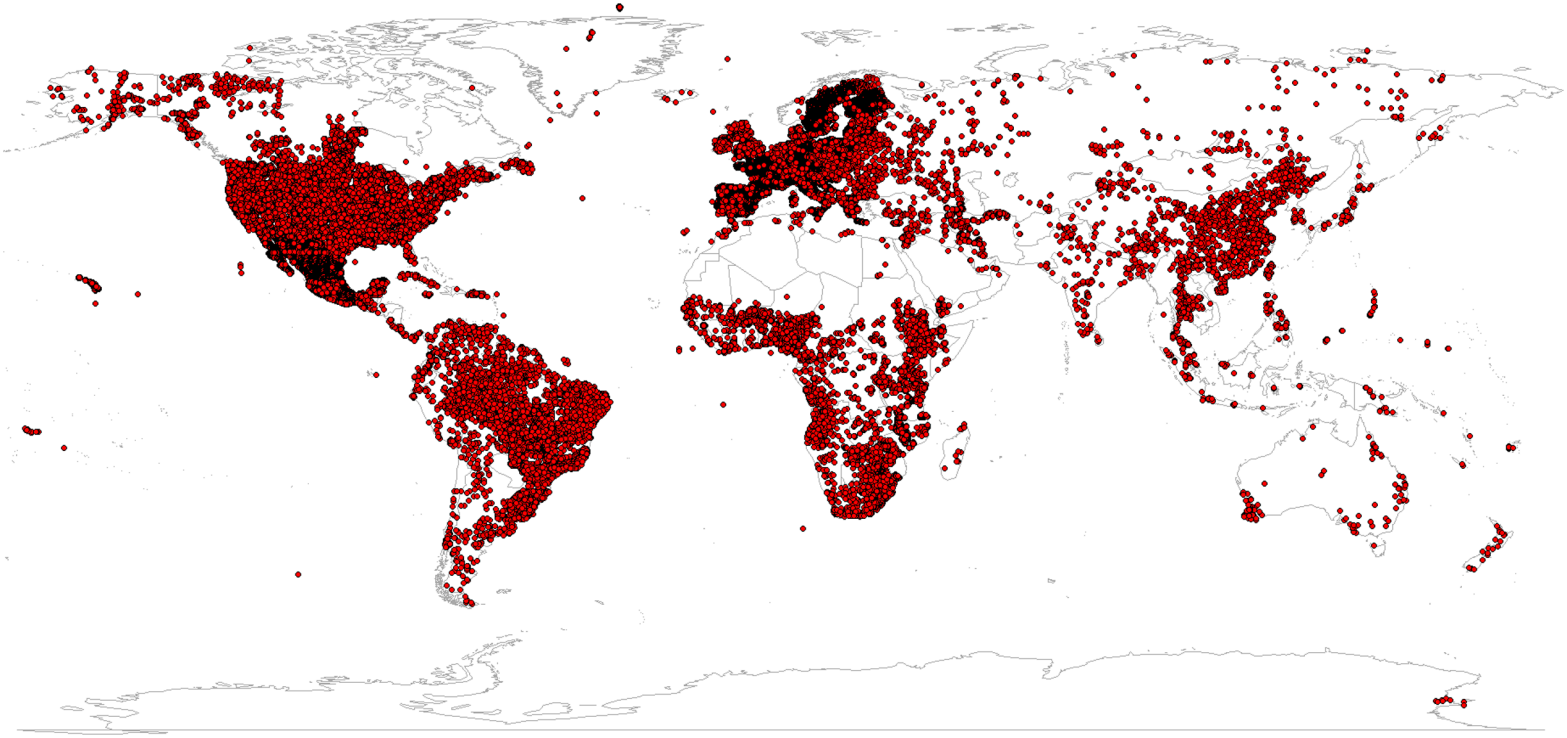
World distribution of soil profiles used to generate the SoilGrids1km product (about 110,000 points). Courtesy of various national and international agencies (see: Acknowledgments).

The compilation of points shown in Figure 2 is possibly the largest compilation of soil ground-truth data in the world. It can be compared, for example, to a compilation of meteorological station data used to generate the WorldClim dataset [29]. A large part of the soil profile data used to generate SoilGrids1km can be accessed via the WorldSoilProfiles.org data portal, however some data sets such as LUCAS [19] have strict data use policies and can only be obtained from the original data provider.

As covariates for SoilGrids1km we used a selection of GIS layers (75): mainly MODIS images, but also climate surfaces [29], Global Lithological Map (GLiM) [30], HWSD mapping units [11], and SRTM DEM-derived surfaces. These layers (apart for the GLiM) are all available via the WorldGrids.org data portal. The actual number of covariates used during the analyses is different for each soil variable as these are iteratively selected for each soil attribute, based on their statistical significance to help predict the specific attribute.

Before model fitting, the original covariates were converted to principal components (*n* = 95) to reduce data overlap and help remove noise and artefacts [7]. Number of components is larger than the number of original covariates because covariates such as lithology and land form classes are converted to indicators before the principal component analysis.

### Soil mask map

**Figure 3:**
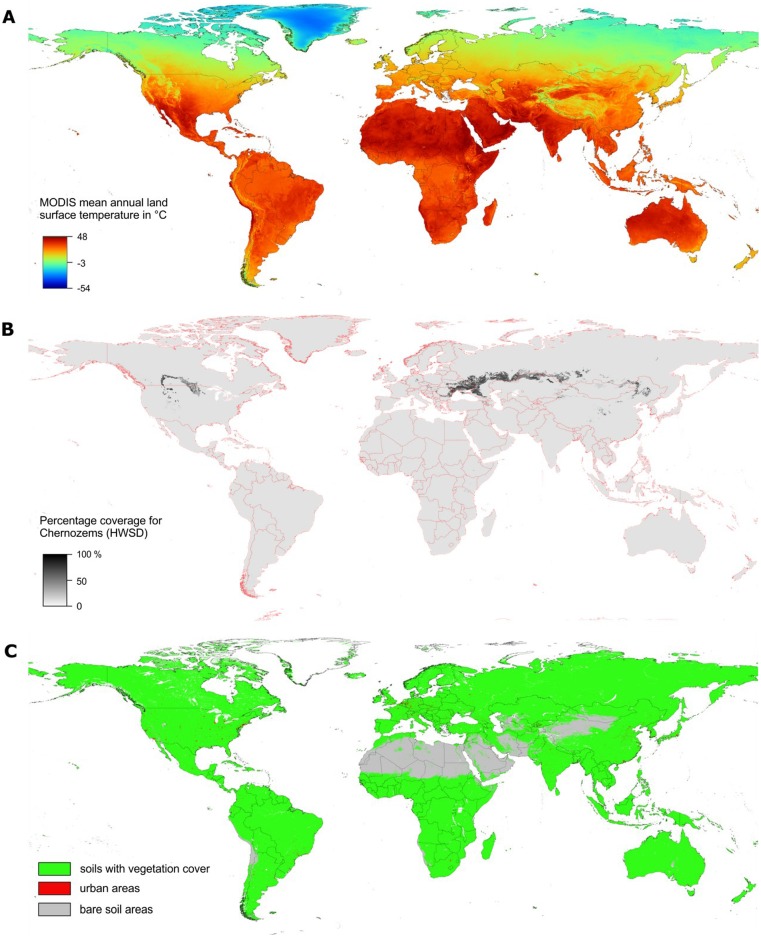
Examples of input layers used to generate SoilGrids1km: (a) long-term day-time MODIS land surface temperature, (b) percent cover Chernozems (based on the HWSD data set), and (c) global soil mask map. The spatial prediction domain of SoilGrids1km are the areas with vegetation cover and urban areas, while bare soil areas have been masked out. See text for more explanation.

We make no spatial predictions for global land cover categories that represent non-active soil areas, such as: artificial surfaces and associated areas (>50% of pixel covered with urban areas), bare rock areas, water bodies [31], shifting sands, permanent snow and ice. The global mask map of soils with vegetation cover and world deserts is shown in Figure 3c.

The soil mask map was derived using the long term MODIS LAI images (MOD15A2), MODIS land cover product (MOD12Q1) [6], and global water mask [31] products. We distinguish three classes in the soil mask:

1. soils with vegetation cover — pixels with MODIS LAI>0 for at least one month in the last 12+ years (2000–2011),
2. urban areas — equal to the MODIS land cover product “*Urban and built-up*” class,
3. bare soil areas — areas without any biological activity but classified as “*Barren or sparsely vegetated*” in the MODIS land cover product.

### Spatial prediction models

**Figure 4:**
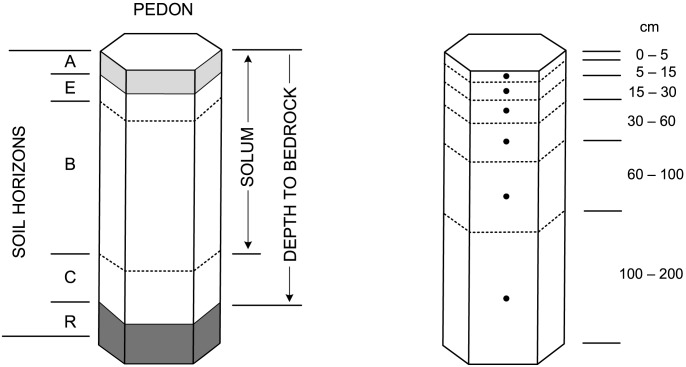
Standard stratification and designation of a soil profile: (left) soil horizons, solum thickness and depth to bedrock (‘R’ layer), and (right) six standard depths used in the *GlobalSoilMap* project [3].

Two groups of spatial prediction models were implemented:

1. 2D or 3D regression and/or regression-kriging [32], [33] combined with splines for numerical properties as implemented in the GSIF package for R. Here, the regression part is fitted using either:
  - Multiple linear regression [34] (for predicting pH, sand, silt and clay percentages and bulk density),
  - General Linear Models (GLM's) with log-link function [35], [36] (for predicting organic carbon content and CEC),
  - Zero-inflated models [37] (for predicting coarse fragments and depth to bedrock; Figure 4),
2. Multinomial logistic regression (as implemented in the nnet package for R) for predicting distribution of soil classes [36].

As a general framework for mapping soil properties and classes we use the regression-kriging method commonly used in geostatistical mapping of soil properties [32], [33], [38]. We extend the existing 2D regression-kriging method to 3D space i.e. to predict values at voxels (Figure 4 right). In addition, we combine regression with splines, so that relationships between the soil property and covariates as well as soil-depth are modelled simultaneously:
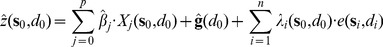
(1)where 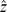 is the predicted soil property, 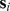 are geographical coordinates, 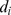 is depth expressed in meters below land surface. Note that 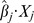 and 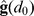 are the trend part of the model, where 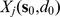 are covariates at the target location 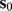 and depth 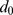, 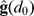 is the predicted vertical trend, modelled by a spline function, and 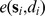 are residuals interpolated using 3D kriging using kriging weights 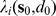. Because all covariates in the current version of SoilGrids1km are in fact 2D (i.e. values available at surface or for top-soil only), we copy the values of covariates for all depths in the regression matrix, which is a simplification. With the increasing availability of gamma radiometrics and similar, we anticipate that also 3D covariates will be used more in the near future with values differing per depth, although many covariates (e.g. elevation) will always remain 2D by definition.

3D regression and/or regression-kriging can be considered novel approaches to modeling soil variation. For comparison, the GlobalSoilMap project (http://globalsoilmap.net) proposes that soil-depth spline functions and spatial prediction functions should be fitted separately [3], [40]. This spatial prediction system can be considered 2.5D because 2D models need to be fitted for each standard depth, i.e. each depth is modelled using a separate model that includes different combinations of covariates and in which data from predictions at one depth do not influence predictions at another. In the case of 3D modelling, a single model (Eq.1) is used for predicting in both *X,Y* and *d* for any property or class of interest, and fitting of the regression equation and residuals occurs at the same time as part of a single step. Another advantage of using a full 3D spatial prediction system, in comparison to the 2.5D, is also that it allows for producing spatial predictions and confidence intervals at any 3D location and not only at standard depths.

**Figure 5:**
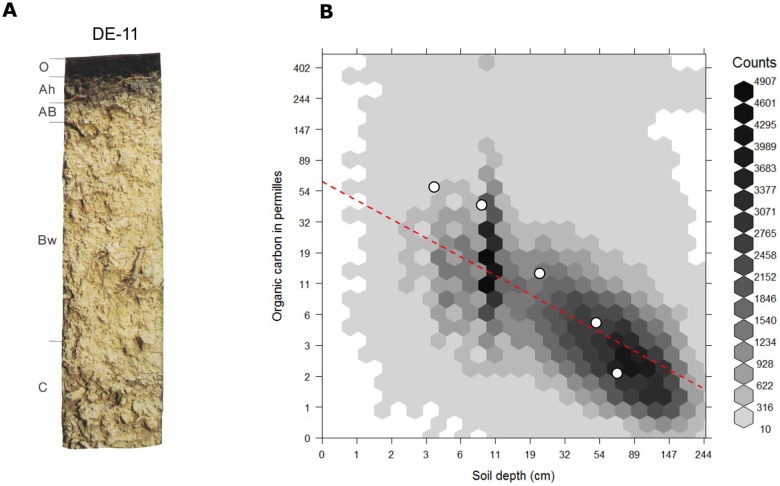
Individual soil profile from the ISRIC soil monolith collection (a) and globally fitted regression model for predicting soil organic carbon using depth only (b). The individual profile horizons are described by Mokma and Buurman [39]. Adjusted R-square for the model on the right is 0.363. Open circles show measured values for the profile on the left.

For each soil property, we have evaluated which version of the model in Eq.(1) would be most applicable. For example, initial tests showed that, for some soil properties e.g. soil organic carbon content and bulk density, the soil-depth relationship (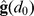) can often be better modelled using a log-log relationship. Consider for example:
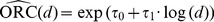
(2)where 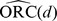 is the predicted soil organic carbon content at depth 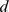 and 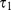 is the rate of decrease with depth. The model fitted using the global compilation of soil profiles (Figure 5b) has 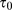 = 4.1517 (standard error 0.005326) and 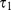 = −0.60934 (standard error 0.00145). This model explains 36% of the variation in the log-transformed ORC, which is a significant portion. This illustrates that any global soil property model can significantly profit from including depth into the statistical modelling. For other soil properties that do not show a monotonic vertical trend, higher order splines implemented via the ns function in the package splines [35] have been used to account for complex, non-linear relationships.

Further, soil covariate layers (*X_j_*) used to produce SoilGrids1km were selected to represent the CLORPT model originally presented by Jenny [38], [41]:
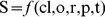
(3)where *S* stands for soil (properties and classes), *cl* for climate, *o* for organisms (including humans), *r* is relief, *p* is parent material or geology and *t* is time. Most of the 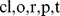 covariates are now publicly available and can be obtained at low cost thanks to NASA's/USGS Earth Observation projects such as MODIS and SRTM. We have also included soil class information (WRB reference groups) extracted from the HWSD (Figure 3b). These are basically traditional soil polygon delineations, comparable to other categorical covariates e.g. land cover classes or geological units.

The 3D regression function used for modelling changes of the of soil organic carbon content in 3D was thus (in R syntax):
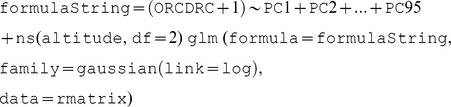
where ORCDRC is the organic carbon content, PC1 to PC95 are the principal components derived from some 75 covariate layers representing Jenny's soil forming factors, altitude is depth in meters from the soil surface, rmatrix is the regression matrix with values of target variable and predictors, ns is the natural spline function and df = 2 sets the number of allowed breakpoints (in this case two breakpoints to allow for curvilinear relationship). Soil classes are useful *‘carriers of soil information’* [42], hence for SoilGrids1km we also provide global predictions for standard soil classes classified according to the two most widely used international soil classification systems:

- FAO's World Reference Base (WRB) — with focus on mapping soil groups e.g. Chernozem, Luvisols, Gleysols and similar. The current system [43] defines 32 reference soil groups.
- United States Department of Agriculture (USDA) Soil Taxonomy — with focus on mapping the soil suborders. The current system [44] defines 67 soil suborders (subdivision of 12 orders: Alfisols, Andisols, Aridisols, Entisols, Gelisols, Histosols, Inceptisols, Mollisols, Oxisols, Spodosols, Ultisols and Vertisols).

Models for predicting WRB soil groups and USDA soil orders were fitted using the nnet package (fits multinomial log-linear models via neural networks) using the default settings of 100 maximum iterations [36]. Soil classes are modeled as 2D variables i.e. the model does not include depth component, e.g.:
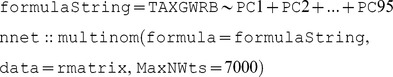
where TAXGWRB is the field observed WRB soil group, nnet::multinom is the function to fit a multinomial logistic regression and MaxNWts sets the maximum allowable number of weights high enough for such a large regression data (regression model with ca. 100 covariates).

Note that all predictions in the initial version of SoilGrids1km were made using regression modelling alone. 3D kriging on a sphere at almost one billion locations (130 million pixels times 6 depths) was beyond our technical capacities in 2013/2014. Efforts to use full 3D regression-kriging to produce the first version of SoilGrids1km were abandoned in response to two main issues. Firstly, the computational load to undertake global kriging was too demanding for the processing resources and time we initially had at our disposal. We are working to both increase our processing power and to make the global kriging algorithms more efficient so we can run them globally for subsequent versions of SoilGrids1km. Secondly, there are very large areas of the world (e.g. Russia, northern Canada) that presently have almost no point profile data. These areas lack a sufficient number and density of point observations to successfully compute residuals, which can then be kriged (otherwise kriging leads to serious artifacts). Since we were unable to produce residuals for large parts of the world, we decided not to try to krige residuals globally at first, at least until we obtain enough new point data to support computing and kriging residuals for all major portions of the globe. A full implementation of the 3D regression-kriging model built for SoilGrids has been run successfully at the continental level in Africa but, for the present (February 2014), we have not been able to apply full 3D regression-kriging globally. As soon as these technical limitations are solved, future versions of SoilGrids1km will likely also include a 3D kriging component.

### Quality control

Resulting spatial predictions in SoilGrids1km are evaluated using two groups of methods:

- Cross-validation: We used 5–fold cross-validation to estimate the average mapping accuracy for each target variable. For continuous soil properties, we evaluate the amount of variation explained by the models [45]; and for soil classes we evaluate the map purity (i.e. proportion of observations correctly classified) and kappa statistic.
- Visual checking and overlay analysis: Because there is a large amount of spatial data, we have requested users to visually explore maps and look for artefacts and inconsistencies. Inconsistencies and artefacts in maps can be continuously reported through a Global Soil Information mailing list.

To derive amount of variation explained by the models for numeric variables we first derive Root Mean Square Error [46]:
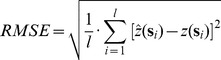
(4)where *l* is the number of validation points. Amount of the variation explained by the model is then:
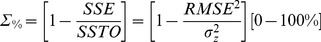
(5)where *SSE* is the sum of squares for residuals at cross-validation points (i.e. 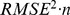), and *SSTO* is the total sum of squares.

### Derivation of secondary soil properties: soil organic carbon stock

**Figure 6:**
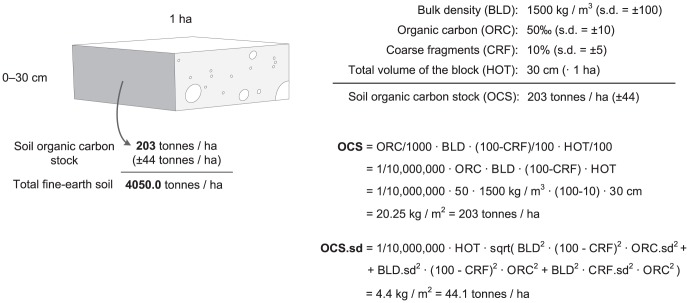
Soil organic carbon stock calculus scheme. Example of how total soil organic carbon stock (OCS) and its propagated error can be estimated for a given volume of soil using organic carbon content (ORC), bulk density (BLD), thickness of horizon (HOT), and percentage of coarse fragments (CRF). See text for more detail.

The SoilGrids1km output maps can be further used for estimation of secondary soil properties which are typically not measured directly in the field and need to be derived from primary soil properties. For instance, consider estimation of the global carbon stock (in t ha^−1^). This secondary soil property can be derived from a number of primary soil properties [47]:
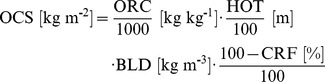
(6)where OCS is soil organic carbon stock, ORC is soil organic carbon mass fraction in permilles, HOT is horizon thickness in cm, BLD is soil bulk density in kg m^−3^ and CRF is volumetric fraction of coarse fragments (>2 mm) in percent (see also Figure 6).

The propagated error of the soil organic carbon stock (Eq.6) can be estimated using the Taylor series method [48]:
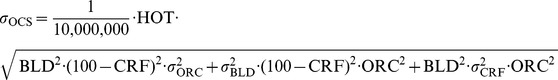
(7)where *σ*_ORC_, *σ*_BLD_ and *σ*_CRF_ are standard deviations of the predicted soil organic carbon content, bulk density and coarse fragments, respectively. Note that we first predict OCS values for all depths/horizons, then aggregate values for the whole profile (0–2 m). We further use a map of predicted depth to bedrock to remove all predictions outside the effective soil depth (areas where soil is shallower than 2 m). A more robust way to estimate the propagated uncertainty of deriving OCS would be to use geostatistical simulations (e.g. derive standard error from a large number of realizations 100) that incorporate spatial and vertical correlations. Because we are dealing with massive data sets, running geostatistical simulations for millions of pixels was not yet considered as an option.

### Software implementation

SoilGrids1km predictions are generated via the GSIF package for R, which makes use of a large number of other basic and contributed packages — gstat, raster, rgdal and other R packages for spatial analysis [49]. GSIF package for R contains most of the functions required to produce SoilGrids, and will remain the main platform in the future to obtain global model parameters and access SoilGrids through an API.

As previously mentioned, the target resolution of SoilGrids1km is relatively coarse, nevertheless, the compu- tational intensity and memory required to produce SoilGrids1km is high: one run of SoilGrids1km takes about 12–16 hours on a 12–core HP Z420 workstation with 64 GiB RAM running on a Windows 7 64-bit system. Note also that since we produce predictions at six depths and uncertainty for each depth, the quantity of GeoTIFF maps produced is in the order of 250×912MiB≈250 GiB. To deal with processing such large data sets we used a combination of tiling and parallel processing, as implemented via the snowfall package for R [50], to maximize the CPU usage and minimize the time required to produce predictions.

The spatial prediction process consists of four main steps:

1. preparation of gridded covariates (principal component analysis),
2. preparation of point data,
3. model fitting and
4. spatial prediction and construction of GeoTiffs.

From the steps listed above, spatial prediction take the longest computing time, which is often in the order of 20 or more hours using the computer specification listed above. As a rule of thumb, we look for mapping frameworks that can generate outputs within 48 hrs. If the whole process from model fitting to prediction and export of maps to GeoTiffs consumes >48 hrs of computing, we consider the system to be impractical for routine operational use.

## Results

### Model fitting

The results of model fitting (Table 1) indicate that the distribution of soil organic carbon content is mainly controlled by climatic conditions, i.e. monthly temperatures and rainfall [51], while the distribution of texture fractions (sand, silt and clay) is mainly controlled by topography and lithology. These key predictors agree with expectations based on existing knowledge. The regression models account for between ca. 20–50% of observed variability in the target variables (Table 1). Detailed model parameters can be obtained from the SoilGrids1km homepage at http://soilgrids.org.

**Table 1:** Mapping performance of SoilGrids1km — amount of variation explained (from 100%) or purity/kappa for categorical variables — for eight targeted soil properties and two soil classes distributed via SoilGrids1km.

Variable name	Type	GSIF code	Units	Range (observed)	Amount of var. explained
Soil organic carbon (dry combustion)	3D	ORCDRC	g kg−1	0–450	22.9%
pH index (H2O solution)	3D	PHIH5X	10−1	2.1–11.0	50.5%
Sand content (gravimetric)	3D	SNDPPT	kg kg−1	1–94	23.5%
Silt content (gravimetric)	3D	SLTPPT	kg kg−1	2–74	34.9%
Clay content (gravimetric)	3D	CLYPPT	kg kg−1	2–68	24.4%
Coarse fragments (volumetric)	3D	GRAVOL	cm3 cm−3	0–89	-
Bulk density (fine earth fraction)	3D	BLDVOL	kg m−3	250–2870	31.8%
Cation-exchange capacity (fine earth fraction)	3D	CEC	cmol+/kg	0–234	29.4%
Depth to bedrock	2D	DBR	cm	0–240	-
Soil group (WRB taxonomy)	2D	TAXGWRB	-	-	28.1% (kappa)
Soil suborder (USDA taxonomy)	2D	TAXOKST	-	-	40.3% (kappa)

WRB = “World Reference Base”; USDA = “United States Department of Agriculture”.

Amount of variation explained by the models (Eq.5) i.e. kappa statistics for soil types was determined using 5–fold cross-validation.

**Figure 7:**
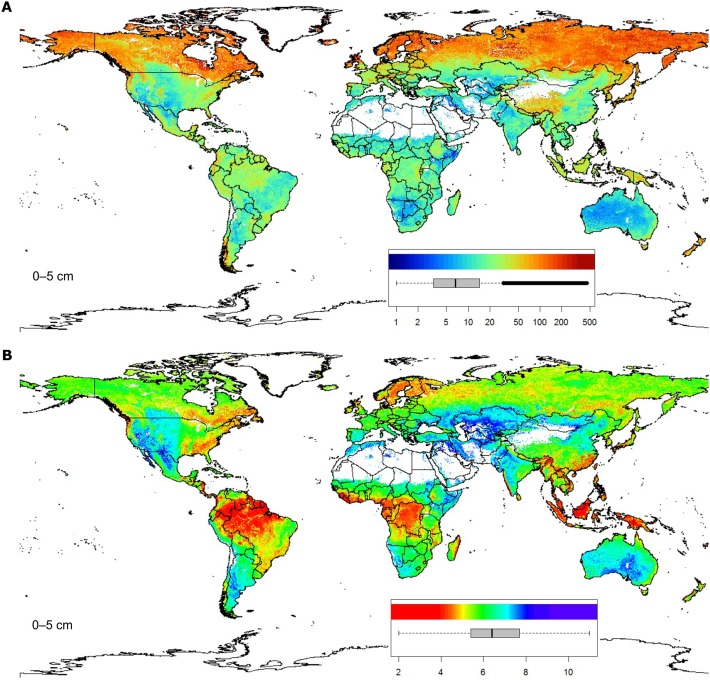
Example of SoilGrids1km layers: (A) soil organic carbon content in permille, and (B) soil pH for the topsoil (0–5 centimetres). Boxplots show the sampled distribution of the soil property based on the present compilation of global soil profile data.

**Figure 8:**
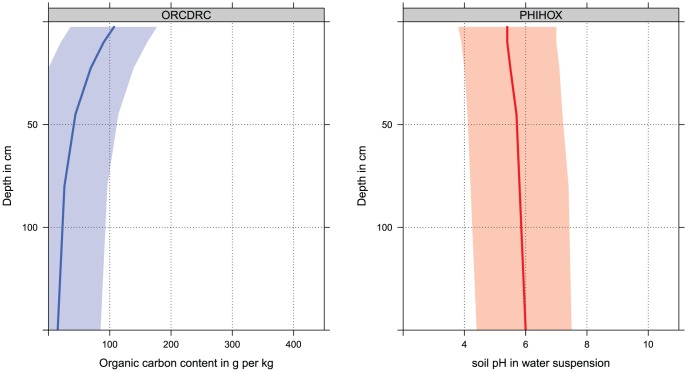
SoilGrids1km-derived soil-depth curves for the profile shown in **Figure 5**. Location of the profile: 6.3831°E, 50.479167°N. The shaded background indicates the 90% prediction interval for each depth. ORCDRC  =  soil organic carbon content in permilles; PHIHOX  =  soil pH in water suspension. See also Table 1.

Figure 7 illustrates two examples of spatial predictions for soil organic carbon content and pH. As mentioned previously, soil organic carbon clearly decreases with depth (see also the soil-depth curves shown in Figure 8). Areas mapped as having elevated values of organic carbon are typically associated with cooler and wetter climate regimes and boreal-tundra type vegetation [51]–[54]. Note that several soil variables have skewed distributions hence also the output predictions are skewed, so that we use log-transformed legends to maximize contrast in the map (Figure 7).

Figure 8 shows predicted values for organic carbon and pH (mean value and confidence intervals) for the same location shown in Figure 5. The prediction intervals are rather wide (see also Figure 11), which is connected to the fact that the models explain only 23–51% of the variation. However, it is important to note that these are global maps of predictions made using relatively coarse resolution covariates. We assume that is unlikely that any effort to map the distribution of soils at a resolution of 1 km could explain a much larger proportion of the total variation in soil properties, as much of this variation occurs over distances less than 1 km [55].

Also note that SoilGrids1km predictions are not capable of representing abrupt changes in values through depth e.g. due to buried horizons, textural heterogeneity or similar. Because we have used linear or close to linear models (plus smoothing splines) to predict values of targeted soil properties and not e.g. regression-trees, these models have smoothed out a significant amount of the variability in the point data, so that it is not realistic to expect abrupt changes in soil properties; at least not vertically (as illustrated previously in Figure 8).

**Figure 9:**
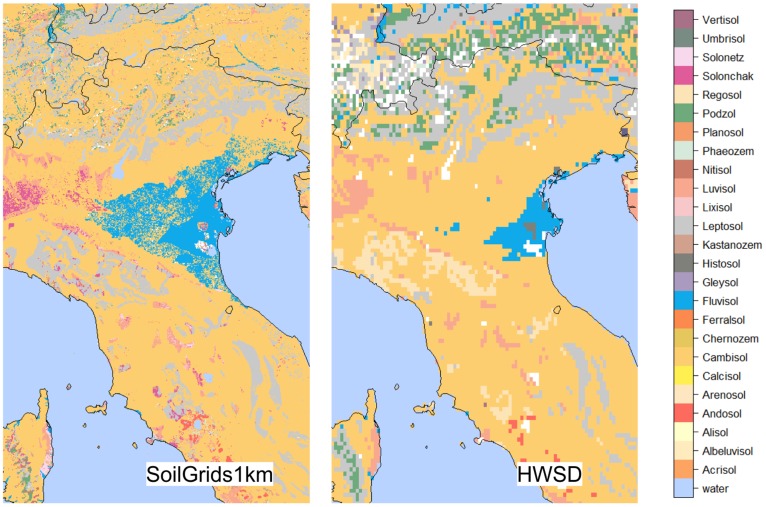
Spatial predictions of WRB soil groups for SoilGrids1km (left) and HWSD data set representing conventional soil maps (right). A zoom in on North of Italy. White pixels indicate missing values.

Figure 9 (with a zoom in on Italy) shows that the SoilGrids1km predictions exhibit an order of magnitude greater spatial detail than previous global soil information products e.g. HWSD. This is mainly because a large stack of fine resolution remote sensing based covariate layers have been used to generate SoilGrids1km, and many of these have shown to be significantly correlated with soil properties and classes. Spatial classification accuracy for mapped soil classes, when evaluated using kappa statistics (Table 1), shows a somewhat better match between what was observed on the ground for the USDA classification system (ground-truth classification available for 16,212 profiles) than for the WRB system (classification available for 37,015 profiles).

For many WRB classes our models predicted occurrences in areas that are inconsistent with a strict definition of geographic areas where these classes can occur. The most difficult to map seem to be WRB classes such as Andosols, Solonchaks, Calcisols and Cryosols. These classes are strictly defined (e.g. Andosols are connected with volcanic activities and specific geology) and we need to explore ways to prepare covariates that will prevent prediction of those classes in areas where, by definition, they should not occur. Likewise, USDA suborders are based on soil moisture and climate regimes, for which we did not currently have global covariate maps, and consequently strictly defined classes such as Xerolls (Mollisols in Mediterranean climate; xeric moisture regime) were predicted in Brazil, which probably does not match the definition of the class.

Multinomial logistic regression is a purely data-driven method, so that the overall mapping performance highly depends on representation of environmental conditions by soil samples. All classes that are poorly represented in the environmental space, due to under-sampling, are understandably difficult to map accurately using a purely data- driven model [56]. Nevertheless, the final results of automated extraction of soil classes using multinomial logistic regression are promising, especially for mapping the USDA classes. The mapping accuracy could probably be improved by adding more classification-related covariates and more field observations of soil taxonomy, hopefully through crowd-sourcing, in areas where the accuracy is critically low.

**Figure 10:**
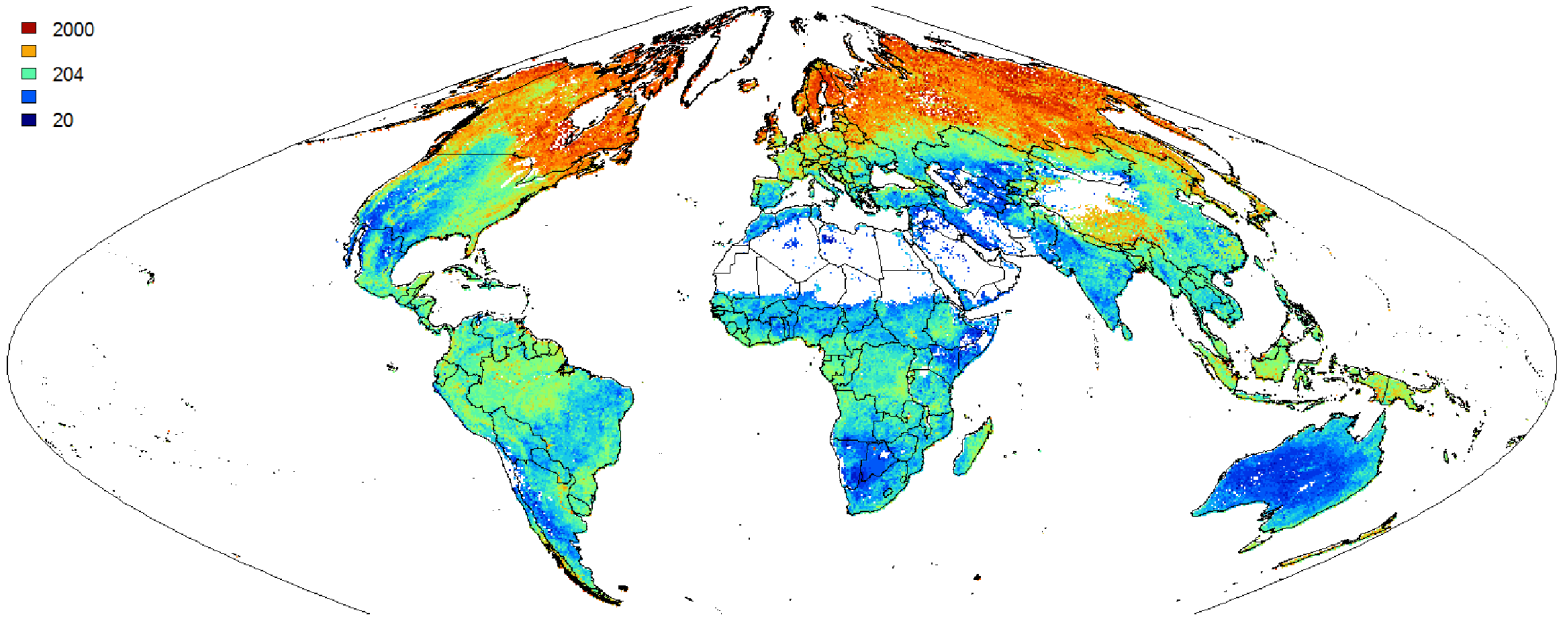
Predicted global distribution of the soil organic carbon stock in tonnes per ha for 0–200 centimetres. Total soil organic carbon stock (here displayed on a log-scale) was estimated as a sum of soil organic carbon stocks for six standard depths and adjusted for the depth to bedrock. Projected in the Sinusoidal equal area projection to give a realistic presentation of areas. Vast deserts (e.g. Sahara or Gobi) can be assumed to contain close to zero organic carbon stock. See also Figure 11.

Figure 10 shows derived total soil organic carbon stock based on Eq.(6). According to this map, the total (baseline) amount of soil organic carbon (up to 2 m depth; excluding deserts, bare rock areas and ice caps) is about 330 t ha^−1^ on average. The highest concentrations of soil organic carbon are in areas of cooler climate and high rainfall, i.e. northern parts of Canada and Russia seem to be pools for most of the world's soil organic carbon. This largely agrees with results by Hugelius et al. [53] and Scharlemann et al. [57].

**Figure 11:**
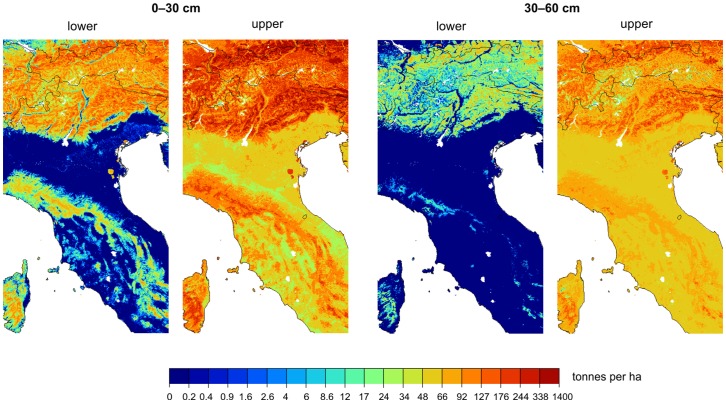
Lower and upper confidence limits (90% probability) of estimated soil organic carbon stock (tonnes per ha) for standard depths 0–30 and 30–60 centimeters for the same area as shown in **Figure 9**. Derived using the procedure explained in Figure 6.

The map shown in Figure 10 can be used to supplement maps of total aboveground biomass (see e.g. Ruesch and Gibbs [58] and Scharlemann et al. [57]). Our results also confirm that, overall, the amount of organic carbon below ground is greater than held in biomass above ground [51].

### Quality issues

The results of cross-validation are shown in Table 1. The cross-validation results, as expected, largely reflect the model fitting success — properties that can be modeled successfully can also be mapped with higher accuracy. The soil properties that were most difficult to map are soil texture fractions, CEC and WRB soil groups. Although the accuracies of the predictions rarely exceed 50% of the total variation, all statistical models are significant showing clear spatial patterns (see e.g. Figure 7). Low cross-validation percentages are common in soil mapping [38], [55], these numbers were not unexpected. Nevertheless, these can be considered promising initial results considering the complexity of harmonization of input point data (see further discussion).

Based on the feedback we received to date from users visiting the project homepage at http://soilgrids. org, the main limitations of SoilGrids1km are:

1. problems arising from poor relationships between covariates and dependent variables e.g. covariates can only explain part of the variability, which could possibly be improved by using more sophisticated statistical models;
2. problems arising from high spatial clustering of sampling locations (see Figure 2; observations are too sparse to improve on the regression using a kriging step);
3. problems associated with using partially-harmonized soil profile data;
4. problems arising from use of HWSD soil mapping units that are of too coarse scale and often not completely harmonized so that the country borders are still visible (obvious artefact);
5. limitations in the usability of SoilGrids1km for spatial planning at county or farm scale due to coarse resolution of the maps;
6. inability to consider and model significant sources of variability e.g. temporal variability due to changes in land use and/or land cover [59];
7. limitations arising from insufficient use of higher quality and finer resolution conventional soil maps prepared at national to regional scales.

## Discussion

SoilGrids1km were released on December 5th 2013 (World Soil Day) at the FAO Rome, as a proposed contribution of the Netherlands to the Global Soil Partnership [60]. The system, at the moment, includes predicted values for (Table 1): soil organic carbon (g kg^−1^), soil pH, sand, silt and clay fractions (%), bulk density (kg m^−3^), cation-exchange capacity (cmol+/kg) of the fine earth fraction, coarse fragments (%), soil organic carbon stock (t ha^−1^), depth to bedrock (in cm; see Figure 4), World Reference Base soil groups [43], and USDA Soil Taxonomy suborders [44]. We focused on generating spatial predictions at six standard depths (0–5 cm, 5–15 cm, 15–30 cm, 30–60 cm, 60–100 cm and 100–200 cm), for which spatially distributed estimates of upper and lower level 90% prediction intervals are presented. As such, we follow the corresponding specifications of the GlobalSoilMap project [3].

Initial predictions of soil classes were made at higher (more general) taxonomic levels for both WRB (soil groups) and Soil Taxonomy (suborders). This was done because the available point profile data sets do not provide a sufficient number of locations representative of all of the lower levels of classification in each system. Without a sufficient number of examples for all lower classes, distributed fully across all of the feature space within which each class can occur, it is not possible to successfully predict many of the lower classes defined for either system. Once we have more point observations that encompass the full range of lower level classes across the entire environmental and geographic spectrum of their distribution, we will be able to predict at a more detailed taxonomic level for both classification systems.

The main purpose of SoilGrids1km is to provide initial, fully worked, examples of how complete and consistent global maps of soil properties, and soil classes, can be produced using currently available legacy soil profile data, freely available gridded maps of global covariates and an on-line automated soil mapping system (GSIF). Additionally, we want to use these initial example maps to implement and demonstrate procedures and systems for supporting free and unrestricted access to what we consider to be the best possible current, globally-complete, estimates of soil properties and soil classes. It is hoped that the production, distribution and use of these new, initial, global soil maps will stimulate additional efforts to both improve these maps and to launch new efforts to collect and use new soils information in new soil mapping and monitoring projects. We especially aim at supporting countries in Africa, and large parts of Asia and Latin America, that often have limited infrastructures to produce soil information at fine resolution [2], [5]. We think that there is a great potential in using the existing field observations and Open Source software to map spatial and spatio-temporal patterns, i.e. without doing any major financial investments.

A number of legitimate concerns exist relative to the initial SoilGrids1km outputs. Probably the most immediate and significant concern has to do with the accuracy and usability of the initial predictions of soil property and class values. We acknowledge that the accuracy of these initial predictions rarely exceeds 50% of the total variation and, for many properties, is often closer to 20–30% (Table 1). The results of cross-validation are informative but need to be taken with caution because most of the soil profiles (Figure 2) were not collected using probability sampling, so that the cross-validation results possibly carry the same sampling bias as the original data [61]. Also note that the accuracy of mapping WRB groups is likely lower than the accuracy of mapping USDA soil suborders because over 40% of the soil profiles that were used for the WRB classification were actually classes translated from national systems. Translation i.e. harmonization of international soil records probably introduces additional noise that cannot be solved by regression modelling.

We argue that it is unreasonable to expect any global map of variation in soil properties to explain much more than 50% of the total observed variation. It is well known that a significant proportion of spatial variation in soil properties occurs over relatively short distances of meters to tens of meters [55], [56]. It is therefore unreasonable to expect that a map of global variation in soil properties, portrayed at a spatial resolution of 1 km, will be able to capture and portray the 50% or more of total variation that occurs at resolutions shorter than 1000 m. Our hope and plan is to gradually improve the accuracy of the predictions by addressing these issues and concerns one by one, in a systematic way (Figure 13). This should be done primarily by working with national and regional soil data agencies, i.e. by adding additional covariates at increasingly finer spatial resolutions and by adding more field/point data from areas that are under-represented.

Although millions of soil profile records have undoubtedly been collected throughout the world, they are often unequally distributed (Figure 2). Likewise, many soil profiles funded by public money are not publicly available or are available in paper format only. Due to unbalanced representation and spatial clustering, predictions in the current version of SoilGrids1km are largely controlled by point data sets available for the USA and Europe. Most of these are from agricultural soils, which inflicts additional bias. Our predictions are therefore likely to exhibit lower accuracy for poorly represented areas such as most of the former Russian Federation, the northern Circumpolar Region, semi-arid and arid areas.

We have also purposely excluded all areas that show no evidence of historical vegetative cover. Our predictions are hence not globally complete. This is a definite drawback for use in global modelling and we acknowledge a need to use either expert judgment or data from other mapping sources to provide alternative predictions for areas with missing values. Again, for deserts and bare rock areas it is perfectly valid to assume a 0 value for soil organic carbon, but it is not as straightforward to estimate soil pH for shifting sand areas for example. For the present, we argue that it is inappropriate to try to make predictions for areas that completely lack vegetative cover e.g. shifting sands of Sahara. These areas have very few to zero point profile observations which can be used to calibrate statistical prediction models. In addition, even if they did have a sufficient number of point profile measurements, the environments of extreme climatic conditions are so different from vegetated ones so that any prediction model is likely to be very different from ones we develop for vegetated areas. We recommend that SoilGrids1km users who require values for the complete land mask fill in the gaps by using expert knowledge or best regional estimates as available from conventional soil mapping (e.g. HWSD, ISRIC-WISE).

It is worth emphasizing that we designed GSIF as a flexible framework with respect to the choice of depths, dimensions (2D or 3D spatial predictions), spatial support size, soil properties and classes and prediction models. Outputs from GSIF are reproducible as a result of use of scripting. Consequently, all maps can be easily updated as new inputs (point and covariate data) become available. We used the GSIF system to generate SoilGrids1km maps for the standard depths defined by the GlobalSoilMap project, but basically one could use the same system for any depth and also for any new property. GSIF is therefore scalable and can be used to produce spatial predictions for virtually any soil property, at any depth and at any spatial or temporal resolution. This, of course, assumes the existence of a sufficient number of point soil observations of appropriate quality and of sufficient covariate layers at sufficiently fine spatial resolution to support modelling at a given spatial resolution.

All methods and models fitted for the purpose of producing SoilGrids1km are available via an Open Source platform (GSIF package for R) and could be adapted for both regional and local mapping. As with input data, the models used to make predictions in GSIF can be improved or replaced in subsequent iterations once better performing models are identified. Prediction models that could be considered in the future include those based on hierarchical Bayes models, regression trees, Random Forests and other machine learning techniques. Regression- trees and similar models could help model better abrupt changes in values vertically, and Random Forests could help emphasize relative importance of specific covariates. The actual modelling approach used to produce any set of predictions will be reviewed continuously to identify and apply the approach that produces the most correct, consistent and usable outputs.

Because the SoilGrids1km maps can be easily updated (or changed) the process used to produce the map (i.e. SoilGrids system) becomes more important than the map itself. Previously, the map product was seen as more important than the process used to produce it, because any map had to be considered as valid and useful for an extended period, as it took so long, and cost so much, to revise or update the map. Under the GSIF model, the final (or most current) map is no longer the most important output and any system that only provides a final map is considered deficient. We hence argue that it is more important to provide access to all data and models needed to produce (and reproduce) the map than to simply provide the final map itself.

In the future, we hope that GSIF will be used by an increasing number and variety of interested parties, including national and regional soil mapping agencies, commercial consulting agencies, advocacy groups and non-governmental organizations. We envisage GSIF as a platform for cooperation, collaboration, innovation and sharing. It will become so if interested parties decide to participate and contribute as committed partners. The number of soil profiles freely shared by the soil science community is constantly growing and national agencies and other data providers are encouraged to contribute their point data to help improve the prediction accuracy locally for specific countries/regions, for the benefit of the global user community and in support of the global UN conventions.

**Figure 12:**
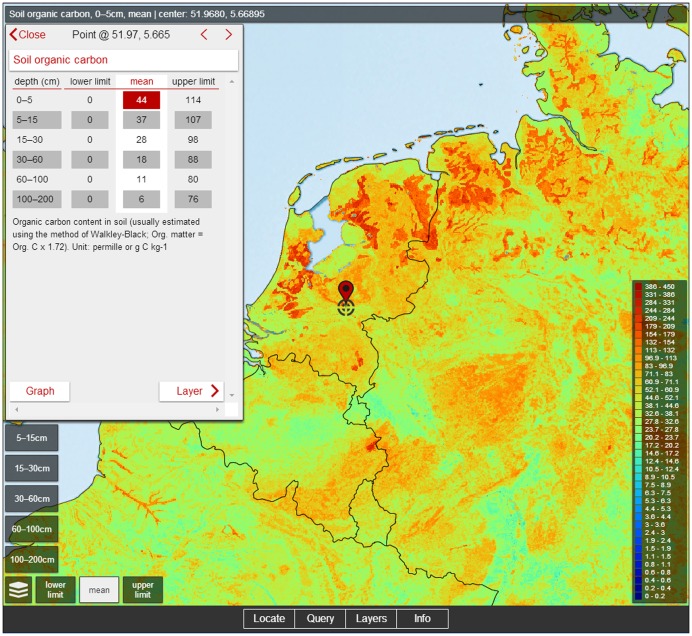
Accessing SoilGrids1km from the SoilInfo app for mobile devices. SoilInfo app is available for download via http://soilinfo.isric.org.

SoilGrids1km are available for download under a Creative Commons non-Commercial license via http://soilgrids.org. SoilGrids1km are also accessible via a Representational State Transfer (http://rest.soilgrids.org) service and via a mobile phone app “SoilInfo App” (http://soilinfo-app.org; Figure 12).

**Figure 13:**
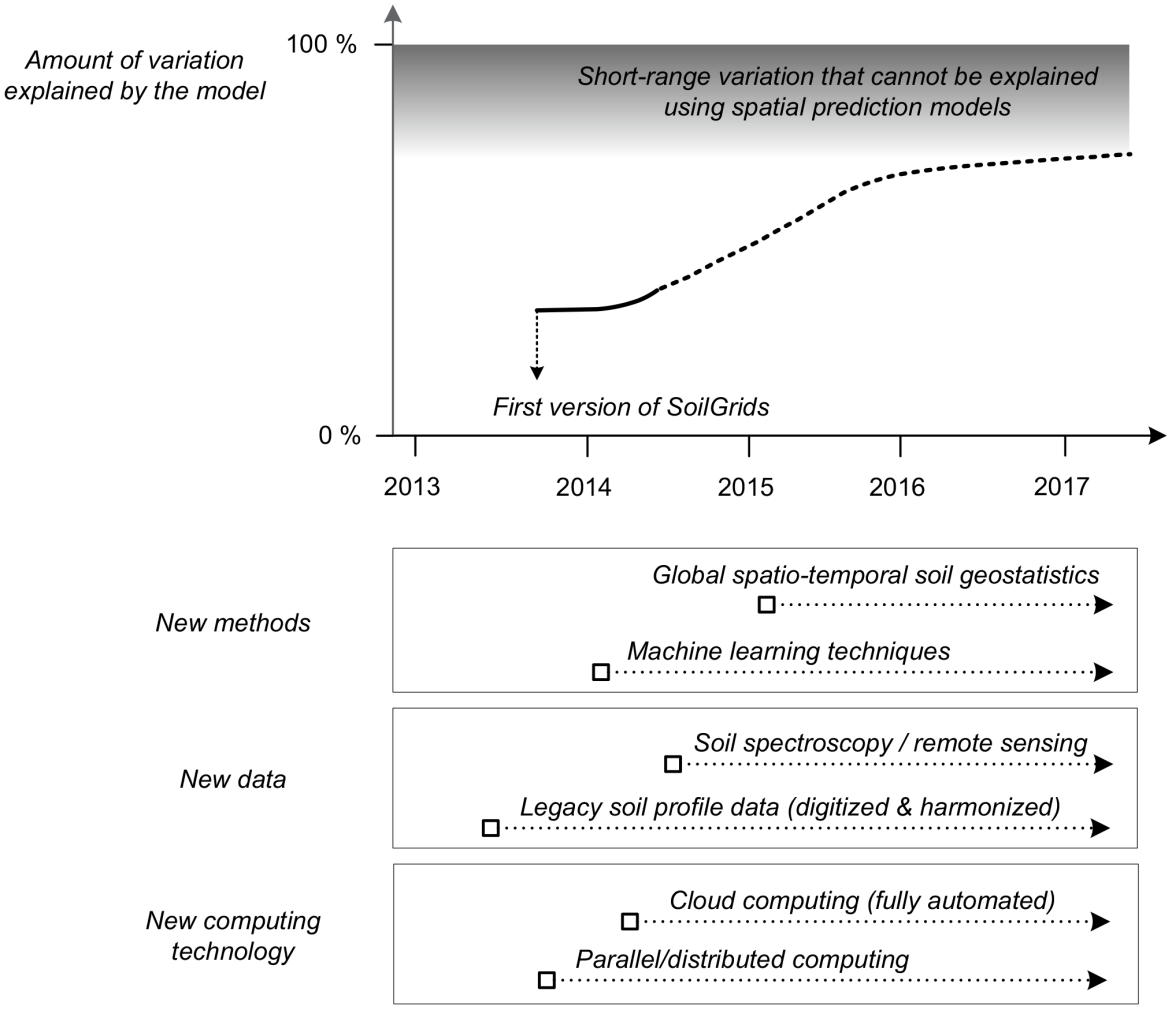
Projected evolution of SoilGrids in the years to come. We anticipate that the main drivers of success of SoilGrids will be use of machine learning methods for model fitting, development of spatio-temporal geostatistical models, use of new sources of field and remote sensing data and use of faster and more powerful computing capacities. Amount of variation explained by these models will eventually reach a *‘natural limit’* (short-range variation that cannot be explained using spatial prediction models), until there is a technological jump in soil remote sensing technology e.g. ground penetrating scanners.
