# Correction: SoilGrids1km — Global Soil Information Based on Automated Mapping

**DOI:** 10.1371/journal.pone.0114788

**Published:** 2014-12-01

**Authors:** The PLOS ONE Staff

There are errors in the legend for Figure 1 “Spatial resolution and temporal coverage/publication time of some widely used global environmental data layers (global soil layers have been highlighted): GLWD — Global Lakes and Wetlands Database, HWSD — Harmonized World Soil Database, MOD12C1 — MODIS Land Cover Type Yearly L3, MOD13C2 — Vegetation Indices Monthly L3, CHLO/SST — MODIS Aqua Level-3 annual Chlorophyll/mid-IR Sea Surface Temperature, FRA — Forest Resources Assessment, GPW — Gridded Population of the World, DMSP-OLS — Nighttime Lights Time Series, GlobCov — Land Cover classes based on the MERIS FR images, GADM — Global Administrative Areas, TanDEM-X — Germany's topographic radar mission.” The complete, correct Figure 1 legend is:

Key agenda setters in the terms of production and dissemination of remote sensing and thematic environmental layers at the beginning of the 21st century include: NASA's MODIS (Moderate-resolution Imaging Spectroradiometer) and Landsat products — in terms of thematic content and usability [6]–[8], and Germany's TanDEM-X new global 12 m resolution DEM with ±2 m vertical accuracy [9]. Based on information retrieved on February 15th 2014.

In the subsection “Spatial prediction models” of the Materials and Methods section (page 6 of the PDF version of the article), the R code snippets should have been divided into two equations. The correct R code snippets are:

formulaString  =  (ORCDRC + 1) ∼ PC1 + PC2 + … + PC95 + ns(altitude, df  =  2)

glm(formula  =  formulaString, family  =  gaussian(link  =  log), data  =  rmatrix)
